# Evaluating Long‐Term Outcomes of Children Undergoing Surgical Treatment for Congenital Heart Disease for National Audit in England and Wales

**DOI:** 10.1161/JAHA.124.035166

**Published:** 2024-10-29

**Authors:** Kate L. Brown, Qi Huang, Ferran Espuny‐Pujol, Julie A. Taylor, Jo Wray, Carin van Doorn, Serban Stoica, Christina Pagel, Rodney C. G. Franklin, Sonya Crowe

**Affiliations:** ^1^ Institute of Cardiovascular Science University College London UK; ^2^ Great Ormond Street Hospital Biomedical Research Centre London UK; ^3^ Clinical Operational Research Unit University College London UK; ^4^ Paediatric Cardiac Surgery, Leeds General Infirmary Leeds UK; ^5^ Paediatric Cardiac Surgery Bristol Children’s Hospital Bristol UK; ^6^ Paediatric Cardiology Royal Brompton and Harefield NHS Foundation Trust London UK

**Keywords:** congenital heart disease, outcomes, pediatric cardiac surgery, transparency, Health Services, Congenital Heart Disease

## Abstract

**Background:**

There is strong interest in the evaluation of longer‐term outcome metrics for congenital heart diseases (CHDs); however, registries focus on postoperative metrics.

**Methods and Results:**

Informed by user online discussion forums and scoping of national data, we selected sentinel CHDs and long‐term outcome metrics suitable for routine monitoring. We then developed sentinel CHD phenotypes and algorithms for identifying treatment pathway procedures using clinical codes. Finally, we calculated the metrics within a retrospective national cohort analysis. The 9 selected sentinel CHDs had a higher‐than‐average prevalence, typically involved surgery in infancy, and were associated with an increased risk of late mortality. The selected metrics of survival and reinterventions at 1, 5, and 10 years were both important and feasible. The cohort included 29 319 (41.3% of all operated CHD births) English and Welsh children born with sentinel CHDs in 2000 to 2022. Example metrics at age 10 years included: survival—hypoplastic left heart syndrome: 57.6% (95% CI, 54.9%–60.4%), functionally univentricular heart: 86.7% (95% CI, 84.6%–88.9%), transposition of the great arteries: 93.1% (95% CI, 92.2%–93.9%), pulmonary atresia: 81.0% (95% CI, 79.1%–82.9%), atrioventricular septal defect: 88.5% (95% CI, 87.5%–89.5%), tetralogy of Fallot: 95.1% (95% CI, 94.4%–95.8%), aortic stenosis: 94.4% (95% CI, 93.3%–95.6%), coarctation: 96.7% (95% CI, 96.2%–97.3%), and ventricular septal defect: 96.9% 95% CI, (96.4%–97.3%); and (2) cumulative incidence of reintervention—hypoplastic left heart syndrome : 54.5% (95% CI, 51.5%–57.3%), functionally univentricular heart: 57.3% (95% CI, 53.9%–60.5%), transposition of the great arteries: 20.9% (95% CI, 19.5%–22.3%), pulmonary atresia: 66.8% (95% CI, 64.2%–69.1%), atrioventricular septal defect: 21.6% (20.3%–23.0%), tetralogy of Fallot: 26.6% (95% CI, 25.2%–28.0%), aortic stenosis: 31.2% (95% CI, 28.8%–33.6%), coarctation: 19.8% (95% CI, 18.6%–21.1%), and ventricular septal defect: 6.1% (95% CI, 5.5%–6.8%).

**Conclusions:**

It is feasible to report important long‐term outcomes of survival and reintervention for sentinel CHDs using routinely collected procedure records, adding value to national audit.

**Nonstandard Abbreviations and Acronyms**
AS: aortic stenosis
AVSD: atrioventricular septal defect
CAG: Confidentiality Advisory Group
FUH: functionally univentricular heart
HLHS: hypoplastic left heart syndrome
HQIP: Healthcare Quality Improvement Partnership
IPCCC: International Pediatric and Congenital Cardiac Code
NCHDA: National Congenital Heart Diseases Audit
NHS: National Health Service
ONS: Office of National Statistics
PA: pulmonary atresia
TGA: transposition of the great arteries
TOF: tetralogy of Fallot
VSD: ventricular septal defect

**Clinical Perspective**

**What Is New?**

- Nine high‐prevalence sentinel congenital heart diseases (CHDs), which always or frequently require early surgery and pose late risks in terms of childhood mortality, were identified as suitable for inclusion in the monitoring of long‐term outcomes.
- The algorithms for identifying these 9 sentinel CHDs, their related CHD subtypes, and their expected surgical treatment pathways were developed for use with national procedure‐registry data.
- Based on the procedure records of 29 319 children with CHD, representing 41.3% of the total number born and starting treatment from England and Wales in the study period of 22 years, the metrics of survival and reinterventions at 1 year, 5 years, and 10 years were selected as important for national audit and calculated for each of the 9 sentinel CHD diagnoses.

**What Are the Clinical Implications?**

- Our study demonstrates the feasibility of reporting long‐term outcomes of children with CHDs, using routinely collected procedure records and national mortality data, thereby advancing the practice of national benchmarking for audit of outcomes.
- Outcome metrics of long‐term survival and reintervention rates, which are highly valued by stakeholders including patient families, provide a fuller picture than outcomes limited in scope to the postoperative period.
- The range is illustrated by example metrics of the 10‐year survival rate, which is lowest for hypoplastic left heart (57.6% [95% CI, 54.9%–60.4%]) and highest for ventricular septal defect (96.9% [96.4%–97.3%]) and the 10‐year cardiac reintervention cumulative incidence involving either surgery or interventional cardiology procedures, which ranged from hypoplastic left heart syndrome (54.5% [95% CI, 51.5%–57.3%]) to ventricular septal defect (6.1% [95% CI, 5.5%–6.8%]).

The postoperative outcomes of pediatric cardiac surgery have been recorded by the National Congenital Heart Diseases Audit (NCHDA) in England since 2000.^1^, ^2^ A report from the Bristol Inquiry found that the 30‐day mortality rate was 13% in children operated on in infancy and 5% in children operated on between the ages of 1 and 15 years from 1991 to 1995.^3^ Postoperative mortality subsequently dropped to 4.3% for all ages combined in 2000, 2.6% in 2010,^1^ and 1.6% in 2019 to 2021.^2^ Over time, children have been undergoing surgical treatments for congenital heart diseases (CHDs) at younger ages and the proportions with complex heart defects and comorbidities have increased.^1^ Patients, clinicians, and service commissioners have therefore aspired to evaluate a wider range of outcome metrics than simply early postoperative survival, to better judge the performance of services and understand the impacts of different types of CHDs on people's lives.^4^, ^5^

The heterogeneity and complexity of the case mix is a barrier to measuring longer‐term outcomes for individual CHD diagnoses from procedure‐based patient registries. CHD is described by thousands of individual cardiac codes,^6^ which, in combination, provide a detailed description of the child's cardiac anatomy and allow for the assignment of a specific “named cardiac diagnosis.”^7^ For many complex CHDs, a series of different cardiac procedures are required over time, with a range of factors and events contributing to longer‐term survival.^8^, ^9^, ^10^ Therefore, despite the challenges, diagnosis‐based analyses of CHD outcomes could provide a more complete picture than procedure‐based analyses for families, clinical teams, and service commissioners. However, there is a lack of consensus about which CHD diagnoses are best suited for long‐term monitoring and which outcomes are most appropriate to measure and over what time frames. In this study we aimed to:

1. Select a set of sentinel CHD diagnoses suited to the measurement and monitoring of long‐term outcomes, and identify feasible long‐term outcome metrics for these sentinel CHDs that are appropriate for future national audit.
2. Define sentinel CHD phenotypes and the expected interventional treatment pathways for each sentinel CHD, and develop algorithms for identifying these within data routinely collected for national audit.
3. Calculate the selected long‐term outcome metrics for each of the selected sentinel diagnoses for England and Wales during the period April 2000 to March 2022.

## METHODS

### Study Design

The study was designed in 3 parts:

1. A scoping analysis of NCHDA data combined with the elicitation of patient, family, and clinician views through qualitative review of transcripts from online discussion forums and structured meetings to select sentinel CHDs and long‐term outcome metrics.
2. A quantitative analysis based on the NCHDA, with survival status from the Office of National Statistics (ONS), to identify sentinel CHD phenotypes and expected interventional treatment pathways and develop algorithms to enable routine classification of each phenotype and outcome metric.
3. A quantitative retrospective cohort analysis based on the same data as (2) to calculate the selected long‐term outcome metrics for each of the sentinel CHD diagnoses for patients in England and Wales born betweeen April 2000 and March 2022 (using the algorithms developed in [2]).

### Approvals

The study was approved by the National Health Service (NHS) Health Research Authority, including the Confidentiality Advisory Group and by the NHS Research Ethics Committee. The research data set is available to researchers only with approvals from these organizations in place. The study was approved by the North of Scotland NHS Research Ethics Committee on February 14, 2020 (research ethics committee number 20/NS/0022), and the Health Research Authority Confidentiality Advisory Group (CAG) on July 12, 2020 (CAG number 20/CAG/0027), which permits the use of registry data for specific research purposes without consent.

The study was approved by the National Institute for Cardiovascular Outcomes Research and the NHS Healthcare Quality Improvement Partnership (HQIP) on December 17, 2020 (application number HQIP 350/20‐CONG‐02). This data sharing agreement was novated to NHS England from June 24, 2022. Approval by NHS Digital (now NHS England) to use civil registration mortality data from the ONS was granted on July 9, 2021.

### Part 1: Selecting Sentinel CHDs and Outcome Metrics

Selection of the sentinel CHD diagnoses and outcome metrics appropriate for national audit was informed by 3 sources of information: parent and patient online discussion forum content; initial scoping of national audit data; and meetings to elicit clinician, analyst, and patient‐family views.

**Online forums**

As we have previously reported,^11^ 3 CHD charities in the United Kingdom individually set up and moderated closed, asynchronous, online discussion groups via their Facebook pages, which were utilized by 343 patients and family members. We noted that the clinical outcomes of long‐term survival and reintervention rates were highly prioritized by patients and families and were also feasible to measure within the national audit data.

2 **Scoping analysis of national audit data**

We performed scoping analysis using NCHDA records of cardiac surgical procedures and interventional cardiology procedures performed in England and Wales from April 2000 to March 2017 and ONS data linked using the patient's NHS number (a unique identifier assigned at birth or first contact with the United Kingdom health system), with life status updated to February 2022. Submission to NCHDA is mandatory and subject to external data validation. Each procedure record contains several diagnostic and procedure codes based on the International Pediatric and Congenital Cardiac Code (IPCCC) schema.^6^ The procedure‐based records that pertain to each individual patient were linked using pseudonymized patient identifiers to create a patient‐based rather than procedure‐based data set. Patient survival was ascertained first using age at death for patients recorded as dead by NCHDA, then age at death from ONS for patients who had a recorded death certificate, and, then, for surviving patients, using the age when their alive status was confirmed by ONS (February 2022). Any patients with missing ONS life status were deemed lost to follow‐up and censored at their most recent discharge age provided by NCHDA. Of note, as the NCHDA is a procedure‐based data set, patients who did not undergo any surgical or interventional cardiac procedures do not appear in the data set.

Patients were assigned to 1 of 24 CHD diagnosis types of varying prevalence and complexity previously defined by Brown et al.^7^ For each of the 24 CHD diagnoses, we ascertained the number of children who started any interventional treatment and the proportion who started treatment before the age of 1 year and, given that long‐term survival and reintervention rates emerged as important to patients and families from the online forums, the proportion who died before the age of 5 years and the proportion who had ≥3 cardiac interventions.

3 **Structured meetings with clinicians, analysts, and patient/family representatives**

Meetings were held with clinicians, analysts, and patient/family representatives in January 2022 and September 2022 (8 and 7 members of the analytical team, 7 and 6 specialist clinicians from cardiology and cardiac surgery, and 3 and 2 patient involvement coinvestigators, respectively) and in November 2022 (9 patient and family advisors: 5 representing user groups and 4 with lived experience of CHD), in which the following questions were discussed:

1. Which CHD diagnoses are most appropriate and feasible to include within national audit (“sentinel” CHD diagnoses)?
2. Which outcome metrics are feasible and appropriate to measure and report within national audit for the sentinel CHD diagnoses?

In March 2023, we held a fourth meeting (9 members of the analytical team, 8 specialist clinicians from cardiology and cardiac surgery, and 1 patient involvement coinvestigator) where we shared our scoping analysis of audit data, sought feedback on proposed outcome metrics, and specifically discussed the following issues (with reference to the 24 CHD diagnoses described in the scoping analysis):

1. The rarest CHDs affected a very small number of children each year nationally, and the small numbers undermine the feasibility of routine monitoring; therefore, these CHD diagnoses should not be selected.
2. The CHDs with higher mortalities and procedure numbers were the most important to include in routine monitoring.
3. Certain CHDs vary widely in severity, with patients receiving surgery at highly variable ages, and NCHDA only captures information for those who have undergone surgery, not for patients with the same diagnosis who have not yet needed surgery. Therefore, for routine monitoring, it is more feasible to focus on the CHD diagnoses that most frequently require an intervention in the first year of life, for which NCHDA will contain all patients who received interventional treatment during their lives and a reasonable period of follow‐up over which to ascertain long‐term outcomes.

### Part 2: Phenotypes of Sentinel CHDs and Their Interventional Treatment Pathways

#### Data Set Inclusion and Exclusion Criteria

We used a data set comprising a retrospective national cohort to develop methods for reporting the outcome metrics for the selected sentinel CHDs. This included NCHDA procedure records for all children who underwent a cardiac surgery or interventional cardiology procedure in England and Wales between April 1, 2000, and March 31, 2022, with life status verified by ONS in August 2023. Records of patients born before April 2000 were excluded to ensure that complete procedure histories were available. Patients from overseas, Scotland, and Northern Ireland were excluded because life status data are collected by ONS for patients from England and Wales only. A small number of records related to patients with major missing or erroneous data were excluded based on clinical review (see the Results section).

#### Sentinel CHDs and Case Mix

Initial broad sentinel CHD diagnoses were identified from within the set of 24 previously defined CHD diagnoses.^7^ Then, informed by clinician views, related literature on CHD complexity^10^, ^12^, ^13^, ^14^ and review of the data set, we refined the phenotype for each sentinel CHD diagnosis. We characterized the phenotypes for important CHD subgroups within each sentinel CHD and defined the complexity characteristics of prematurity (birth at gestation <37 weeks) and congenital noncardiac conditions^15^ using diagnostic and procedure codes. We ascertained the recorded age and weight at first cardiac intervention for each child.

#### Defining Expected Interventional Treatment Pathways

Considering clinical views, related literature on interventional CHD treatment,^9^, ^16^, ^17^, ^18^, ^19^, ^20^, ^21^, ^22^, ^23^, ^24^, ^25^, ^26^, ^27^ data summaries for each CHD, and samples of unusual procedure histories, we defined the expected interventional treatment pathways in terms of cardiac surgery, interventional catheters, and hybrid types where applicable, for each sentinel CHD based on diagnosis and procedure codes. There are 2 broad groups of cardiac interventional procedures: “reparative” (in which an attempt is made to correct the heart defect in biventricular CHDs) and “palliative” (in which a noncorrective procedure is undertaken in CHDs where repair is infeasible). For functionally single‐ventricle CHDs (eg, HLHS), the expected treatment pathway consists of a series of exclusively palliative procedures: stage 1 procedures, stage 2 Glenn surgery, and stage 3 Fontan‐type completion.^28^ Our previously defined interventional treatment pathways for functionally single‐ventricle heart disease^10^, ^12^, ^29^ were used to identify the expected treatment pathway for the selected sentinel CHDs with functionally single‐ventricle circulation. In biventricular CHDs (eg, TOF), the expected treatment pathway involves a reparative surgery and potentially also a palliative stage 1 procedure, usually undertaken in small babies to enable their circulation to support them until they grow large enough for a reparative procedure, eg, systemic to pulmonary arterial shunt in TOF followed by repair at a later procedure.^17^ We characterized the reparative surgeries and first‐stage procedures for each selected biventricular sentinel CHD. For CHDs where a range of anatomy occurs and the treatment pathway may be either a biventricular repair or staged single‐ventricle palliation (eg, in AVSD, since the CHD may present with ventricles that are either balanced or unbalanced in size),^30^ the treatment pathways were defined involving both palliative and reparative procedures. Finally, treatment pathways may involve a “prepathway procedure,” which is a short‐term intervention that occurs after the child's birth and before the first staged surgery (eg, balloon atrial septostomy in TGA), which we defined drawing on peer‐reviewed definitions from prior studies.^10^, ^12^, ^28^

#### Patients With Suspected Missing Data

We identified patients with suspicious missing or unusual procedure sequences for the CHD diagnoses, informed by clinical expertise. In future routine monitoring, all such patients will be flagged with the treating centers for correction.

#### Rules for Use in Routine Reporting

We developed rules for assigning the sentinel diagnoses, subgroups, complexity characteristics, treatment pathways, and suspected missing data to individual patients using the procedure and diagnosis data routinely collected for national audit to enable routine monitoring of outcomes by sentinel diagnosis. These rules are available at https://www.ucl.ac.uk/clinical‐operational‐research‐unit/research‐domains/congenital‐heart‐disease‐children‐and‐adults (in the “CHAMPION” dropdown list). When we assigned record‐level diagnosis and procedure codes, each child could have only one of each defined pathway procedure applicable to the CHD diagnoses, ie, one occurrence of a stage 1, stage 2, stage 3, or reparative procedure per patient.

### Part 3: Calculating the Outcome Metrics by Sentinel CHD for Patients in England and Wales From April 2000 to March 2023

#### Cohort Population

The cohort population was as defined in Methods Part 2a.

#### Outcome Metrics and Analysis

Survival was ascertained as described in Methods Part 1b and calculated at 1, 5, and 10 years of age.

Reinterventions were defined as cardiac surgery of open and closed types, interventional catheterizations, electrophysiology interventions, or hybrid procedures undertaken for residual, recurrent, or acquired cardiac conditions over and above the expected treatment pathway as defined for each sentinel CHD in Method Part 2c. Pathway procedures were identified first, and any subsequent occurrence of a defined pathway procedure, ie, a reoperation, was classified as a reintervention. Reinterventions were grouped as surgical and interventional cardiology types and were calculated at 1, 5, and 10 years of age.

For each sentinel CHD diagnoses, we estimated survival rate over time using Kaplan–Meier approach and calculated the cumulative incidence of reintervention over time using cumulative incidence functions, taking account of death and heart transplant without reintervention as competing events.

Data management were performed with Stata 15 software (StataCorp LLC) and statistical analyses were performed using R version 4.3.0 (Foundation for Statistical Computing).

## RESULTS

### Part 1: Sentinel CHD Diagnoses

Table 1 shows, for each of the 24 candidate CHD diagnoses based on Brown et al,^7^ the number of patients, the proportion of patients starting interventional treatment in infancy, the proportion with ≥3 cardiac procedures, and the proportion who died before 5 years old. CHD diagnoses excluded based on the low number of patients, low proportion starting treatment in infancy, or low event rates are highlighted.

**Table 1:** Selection of Sentinel CHD Diagnoses

Primary diagnosis	Patients, n	Proportion starting interventional treatment in infancy, %	Proportion with 3+ cardiac procedures, %	Proportion died before age 5 y, %
Diagnoses selected for inclusion in the cohort				
HLHS	1393	98.9	64.0	37.4
FUH	1327	92.4	65.3	19.9
TGA all complex types	2015	97.6	28.8	7.8
TGA and intact ventricular septum	1165	99.0	10.9	5.1
Pulmonary atresia and intact ventricular septum	410	98.3	54.4	23.4
Pulmonary atresia and VSD	1230	86.4	55.0	19.1
AVSD	2884	71.4	9.1	9.2
TOF including DORV	3290	81.3	14.9	4.4
Aortic valve stenosis	1273	75.6	19.5	7.9
Aortic arch obstruction ± VSD or ASD	3297	84.8	9.3	3.8
VSD	5081	79.9	4.2	3.3
Diagnoses excluded: low number of patients, low proportion starting treatment in infancy, or low event rates				
Mitral valve diseases	764	52.7	13.4	7.7
Subaortic stenosis (isolated)	314	18.8	6.1	0.6
Aortic regurgitation	313	16.9	2.9	0.6
Atrial septal defect	3891	13.4	1.3	1.6
Patent ductus arteriosus	8928	56.9	0.5	6.0
Diagnoses excluded: borderline for reasons of low number of patients, low proportion starting treatment in infancy, or low event rates				
Common arterial trunk	443	97.1	41.3	17.8
Interrupted aortic arch	339	95.3	33.6	13.9
Tricuspid valve including Ebstein anomaly	423	61.2	16.5	9.2
Totally anomalous pulmonary venous connection	701	96.6	3.7	8.7
Pulmonary stenosis	2660	67.1	3.8	2.4
Diagnoses excluded: mixed group of very rare conditions				
Miscellaneous primary congenital diagnoses	1951	71.9	22.5	10.4

The selection of sentinel congenital heart disease (CHD) diagnoses was based on National Congenital Heart Diseases Audit data from April 2000 to March 2017. AVSD indicates atrioventricular septal defect; DORV, double‐outlet right ventricle; FUH, functionally univentricular heart; HLHS, hypoplastic left heart syndrome; TGA, transposition of the great arteries; TOF, tetralogy of Fallot; and VSD, ventricular septal defect.

The following sentinel CHD diagnoses were selected (in order of decreasing clinical complexity), with clinician‐defined important subgroups shown in parentheses: hypoplastic left heart syndrome (HLHS), functionally univentricular heart (FUH) (double‐inlet ventricle and tricuspid atresia), transposition of the great arteries (TGA) (TGA with intact septum, complex TGA with or without pulmonary stenosis), pulmonary atresia (PA) (PA with or without ventricular septal defect [VSD]), atrioventricular septal defect (AVSD) (complete, partial, unbalanced and tetralogy with AVSD), tetralogy of Fallot (TOF) (with or without double‐outlet right ventricle), valvar aortic stenosis (AS; with or without other levels of left heart obstruction), coarctation of the aorta (with or without VSD), and VSD (single or multiple). Although a common condition involving early intervention and late mortality, we excluded persistent or patent ductus arteriosus, because this condition is related to prematurity not CHD for most children.

Of the 9 sentinel CHDs selected, VSD was the least severe and most prevalent condition, in 5081 children, of whom 79.9% were operated on in infancy, 4.2% had ≥3 cardiac interventions, and 3.3% died before 5 years old. The most severe diagnosis was HLHS in 1393 children, of whom 98.9% had at least 1 operation in infancy, 64% had ≥3 cardiac interventions, and 37.4% died before the age of 5 years.

#### Outcome Metrics

The outcome metrics selected were survival to the ages of 1 year, 5 years, and 10 years, and the cumulative incidence of children with at least 1 additional surgery and additional therapeutic catheterization as a reintervention by the ages of 1 year, 5 years, and 10 years.

### Part 2: Cohort Population

The data set incorporated all procedures in NCHDA undertaken in 71 050 children born between April 2000 and March 2022 (Figure S1 depicts the inclusion flow chart). We excluded 2233 children outside England and Wales, as well as 1411 non‐NHS patients, typically foreign nationals, for whom reliable life status could not be ascertained. After excluding 263 patients who had no cardiac procedure codes and 86 who had major data errors, there were 67 406 children from England and Wales in the data set. A total of 29 319 (41.3%) patients had one of the sentinel CHDs.

#### Sentinel CHDs, CHD Subgroups, and Comorbidities

Table 2 shows the number of children with each defined sentinel CHD and each CHD subgroup. For example, there were 4358 children with AVSD consisting of tetralogy with AVSD (n=215), unbalanced AVSD (n=291), partial AVSD (n=1108), and complete AVSD (n=2744). The median number of children who started treatment per year at each center was HLHS: 14 (interquartile range [IQR], 8–17), FUH: 10 (IQR, 7–17), TGA: 48 (IQR, 37–56), PA: 18 (IQR, 14–23), AVSD: 36 (IQR, 28–50), TOF: 44 (IQR, 41–50), AS: 12 (IQR, 9–14), coarctation: 51 (IQR, 46–62), and VSD: 63 (IQR, 50–76).

**Table 2:** Number of Patients in Each CHD Diagnosis Group

Diagnosis	Diagnosis subgroup	Whole cohort using data from April 2000 to March 2022							Last 3 y (April 2019–March 2022)	
		Patients, n	Patients with suspected missing/miscoded data, %	Average annual number	Age (d since birth) at first cardiac procedure media,n (IQR)	Weight (kg) at first cardiac procedure media, n (IQR)	Number of preterm births, %	Patients with congenital comorbidities, %	Patients, n	Patients per center, median (IQR)
Diagnoses that are exclusively single ventricle										
HLHS	HLHS	1296	68 (5.2%)	59	4 (3–6)	3.1 (2.8–3.5)	54 (4.2%)	182 (14.0%)	135	14 (8–17)
FUH	Total (FUH)	997	5 (0.5%)	45	18 (6–69)	3.4 (3.0–4.3)	78 (7.8%)	145 (14.5%)	119	10 (7–17)
	Double‐inlet ventricle	443	4 (0.9%)	20	15 (6–70)	3.5 (3.0–4.4)	38 (8.6%)	57 (12.9%)	48	4 (2–7)
	Tricuspid atresia	554	1 (0.2%)	25	22 (6–68)	3.3 (2.9–4.2)	40 (7.2%)	88 (15.9%)	71	6 (4–11)
Diagnoses with a primary diagnosis that can be either managed by single‐ventricle or biventricular pathway										
TGA	Total (TGA)	3838	79 (2.1%)	174	5 (1–13)	3.3 (3.0–3.7)	156 (4.1%)	201 (5.2%)	453	48 (37–56)
	Complex TGA and PS	447	24 (5.4%)	20	13 (3–55)	3.5 (3.0–4.1)	32 (7.2%)	51 (11.4%)	36	3 (2–5)
	Complex TGA without PS	1283	26 (2.0%)	58	8 (2–17)	3.3 (3.0–3.7)	44 (3.4%)	91 (7.1%)	171	16 (15–20)
	TGA with intact ventricular septum	2108	29 (1.4%)	96	2 (1–8)	3.3 (3.0–3.7)	80 (3.8%)	59 (2.8%)	246	28 (20–30)
PA	Total (PA)	1643	1 (0.1%)	75	14 (5–84)	3.3 (2.8–4.3)	175 (10.7%)	462 (28.1%)	187	18 (14–23)
	PA and VSD	1131	None	52	34 (8–148)	3.4 (2.8–5.7)	140 (12.4%)	406 (35.9%)	122	11 (9–15)
	PA with intact ventricular septum	512	1 (0.2%)	23	5 (3–10)	3.2 (2.8–3.6)	35 (6.8%)	56 (10.9%)	65	6 (5–7)
AVSD	Total (AVSD)	4358	77 (1.8%)	198	157 (94–430)	5.3 (4.1–8.5)	328 (7.5%)	2345 (53.8%)	402	36 (28–50)
	Tetralogy AVSD	215	11 (5.1%)	10	158 (60–342)	5.5 (3.7–8.0)	22 (10.2%)	161 (74.9%)	17	2 (1–2)
	Unbalanced AVSD	291	19 (6.5%)	13	40 (9–140)	3.5 (2.9–5.0)	33 (11.3%)	120 (41.2%)	32	3 (2–5)
	Partial AVSD	1108	None	50	770 (306–1402)	11.4 (7.3–15.3)	44 (4.0%)	347 (31.3%)	53	4 (2–8)
	Complete AVSD	2744	47 (1.7%)	125	133 (90–192)	4.8 (4.0–6.0)	229 (8.3%)	1717 (62.6%)	300	24 (22–41)
Diagnoses that are exclusively biventricular										
TOF	Total (TOF)	4643	75 (1.6%)	211	193 (107–306)	6.7 (5.0–8.2)	410 (8.8%)	1036 (22.3%)	465	44 (41–50)
	Tetralogy with absent pulmonary valve	185	2 (1.1%)	8	146 (45–370)	5.6 (3.7–8.4)	14 (7.6%)	75 (40.5%)	10	1 (1–1)
	Tetralogy with DORV	546	19 (3.5%)	25	156 (56–279)	5.8 (3.6–7.7)	84 (15.4%)	185 (33.9%)	68	8 (4–10)
	Standard tetralogy	3912	54 (1.4%)	178	198 (119–306)	6.8 (5.3–8.3)	312 (8.0%)	776 (19.8%)	387	40 (30–43)
AS	Total (AS)	1631	None	74	112 (19–1305)	5.9 (3.6–15.7)	93 (5.7%)	141 (8.6%)	122	12 (9–14)
	AS and mutilevel left‐sided heart obstruction	434	None	20	45 (7–649)	4.1 (3.2–10.6)	34 (7.8%)	63 (14.5%)	33	3 (2–4)
	Isolated AS	1197	None	54	141 (29–1706)	6.7 (3.9–17.7)	59 (4.9%)	78 (6.5%)	89	8 (6–11)
Coarctation	Total (coarctation)	4338	None	197	20 (8–110)	3.6 (3.0–5.5)	315 (7.3%)	521 (12.0%)	515	51 (46–62)
	Coarctation plus VSD	1333	None	61	13 (7–40)	3.3 (2.8–3.8)	112 (8.4%)	199 (14.9%)	198	18 (16–26)
	Isolated coarctation	3005	None	136	34 (9–255)	3.9 (3.1–8.0)	203 (6.8%)	322 (10.7%)	317	32 (29–36)
VSD	Total (VSD)	6575	None	299	154 (94–317)	5.3 (4.2–7.6)	658 (10.0%)	1565 (23.8%)	688	63 (50–76)
	Multiple VSDs	426	None	19	105 (66–178)	4.3 (3.4–5.5)	65 (15.3%)	102 (23.9%)	46	4 (3–6)
	Isolated VSD	6149	None	280	158 (97–335)	5.4 (4.2–7.9)	593 (9.6%)	1463 (23.8%)	642	60 (44–72)

AS indicates aortic stenosis; AVSD, atrioventricular septal defect; CHD, congenital heart disease; DORV, double‐outlet right ventricle; FUH, functionally univentricular heart; HLHS, hypoplastic left heart syndrome; IQR, interquartile range; PA, pulmonary atresia; PS, pulmonary stenosis; TGA, transposition of the great arteries; TOF, tetralogy of Fallot; and VSD, ventricular septal defect.

Congenital comorbidities affected: HLHS: 14.0%, FUH: 14.5%, TGA: 5.2%, PA: 28.1%, AVSD: 53.8%, TOF: 22.3%, AS: 8.6%, coarctation: 12.0%; and VSD: 23.8%.

Premature birth affected HLHS: 4.2%, FUH: 7.8%, TGA: 4.1%, PA: 10.7%, AVSD: 7.5%, TOF: 8.8%, AS: 5.7%, coarctation: 7.3%; and VSD: 10.0%.

Median patient age (days since birth) and weight (kg) at first cardiac procedure were HLHS: 4 days (IQR, 3–6 days) and 3.1 kg (IQR, 2.8–3.5 kg), FUH: 18 days (IQR, 6–69 days) and 3.4 kg (IQR, 3.0–4.3 kg), TGA: 5 days (IQR, 1–13 days) and 3.3 kg (IQR, 3.0–3.7 kg), PA: 14 days (IQR, 5–84 days) and 3.3 kg (IQR, 2.8–4.3 kg), AVSD: 157 days (IQR, 94–430 days) and 5.3 kg (IQR, 4.1–8.5 kg), TOF: 193 days (IQR, 107–306 days) and 6.7 kg (IQR, 5.0–8.2 kg), AS: 112 days (IQR, 19–1305 days) and 5.9 kg (IQR, 3.6–15.7 kg), coarctation: 20 days (IQR, 8–110 days) and 3.6 kg (IQR, 3.0–5.5 kg), and VSD: 154 days (IQR, 94–317 days) and 5.3 kg (IQR, 4.2–7.6 kg), respectively.

#### Expected Interventional Treatment Pathways

All patients with HLHS and FUH had a single‐ventricle pathway. There was a single‐ventricle surgical pathway in 2.4% of 3759 patients with TGA, 10.2% of 1642 patients with PA, and 3.1% of 4280 patients with AVSD; the remaining children had a biventricular pathway. Among children with TOF, VSD, coarctation, and AS, all patients (except 5 with TOF) had a biventricular pathway.

**Figure 1:**
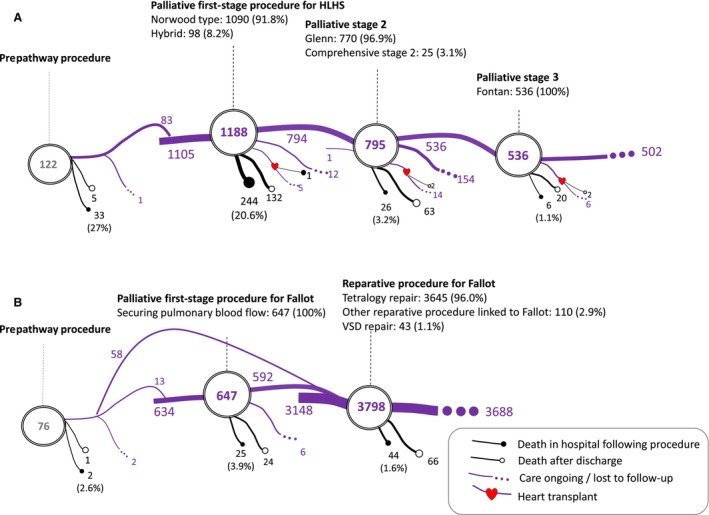
Pathway diagram for congenital heart disease diagnoses. (**A**) Hypoplastic left heart syndrome (HLHS; n=1228) and (**B**) standard tetralogy (n=3858). Patients with suspected missing or miscoded data (68 HLHS and 54 standard tetralogy) were removed from the pathway diagram. The percentage of the pathway procedure subtype was computed based on patients who had the pathway procedure. VSD indicates ventricular septal defect.

Figure 1 depicts 2 example treatment pathways: (1) for the 1228 children with HLHS (a single‐ventricle condition); and (2) for the 3858 patients with standard TOF (the main CHD subgroup of this biventricular condition). For HLHS, 1188 (96.7%) patients had undergone stage 1 palliation; 795 (64.74%) had undergone stage 2 palliation; and 536 (43.6%) had undergone a Fontan. For standard TOF, 647 (16.8%) patients had a stage 1 palliative procedure and 3798 (98.4%) had a reparative procedure.

#### Patients With Suspected Missing Data

Of the whole study cohort 29 319, 305 (1.0%) patient records were flagged as containing potential missing or miscoded data.

### Part 3: Cohort Population

The cohort population is described in Results Part 2a.

#### Outcome

**Figure 2:**
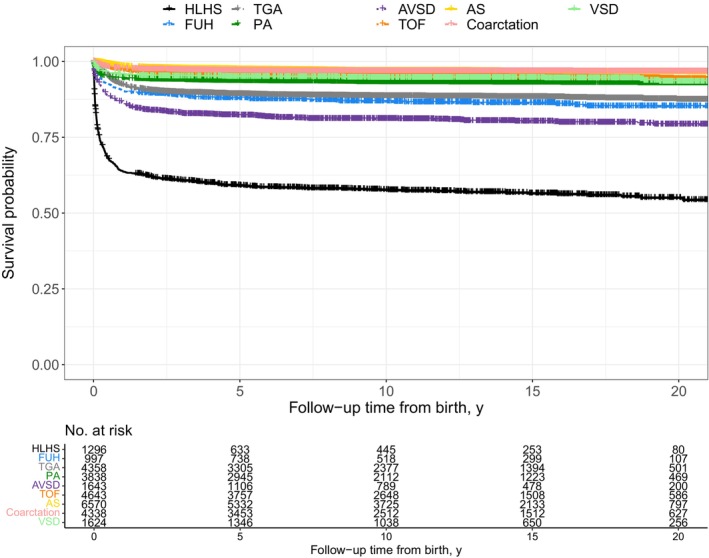
Kaplan–Meier survival curves for each congenital heart disease diagnosis. AS indicates aortic stenosis; AVSD, atrioventricular septal defect; FUH, functionally univentricular heart; HLHS, hypoplastic left heart syndrome; PA, pulmonary atresia; TGA, transposition of the great arteries; TOF, tetralogy of Fallot; and VSD, ventricular septal defect.

Table 3 and Figure 2 shows survival metrics for each sentinel CHD diagnosis. For example, at 10 years of age, the survival rate was: HLHS: 57.6% (95% CI, 54.9%–60.4%), FUH: 86.7% (95% CI, 84.6%–88.9%), TGA: 93.1% (95% CI, 92.2%–93.9%), PA: 81.0% (95% CI, 79.1%–82.9%), AVSD: 88.5% (95% CI, 87.5%–89.5%), TOF: 95.1% (95% CI, 94.4%–95.8%), AS: 94.4% (95% CI, 93.3%–95.6%), coarctation: 96.7% (95% CI, 96.2%–97.3%), and VSD: 96.9% (95% CI, 96.4%–97.3%).

**Table 3:** Survival Metrics in Each CHD Diagnosis Group

Diagnosis	Diagnosis subgroup	Follow‐up time, y	Survival rates with 95% CI		
		Median (IQR) [minimum, maximum]	At age 1 y	At age 5 y	At age 10 y
Diagnoses that are exclusively single ventricle					
HLHS	HLHS	4.7 (0.2–12.8) [0, 23.4]	63.8% (61.3%–66.5%)	59.1% (56.4%–61.8%)	57.6% (54.9%–60.4%)
FUH	Total (FUH)	10.5 (4.7–16.1) [0, 23.4]	90.4% (88.5%–92.2%)	87.8% (85.8%–89.9%)	86.7% (84.6%–88.9%)
	Double‐inlet ventricle	11.0 (5.5–16.0) [0, 23.4]	93.7% (91.4%–96.0%)	91.3% (88.6%–94.0%)	90.0% (87.1%–92.9%)
	Tricuspid atresia	10.0 (3.9–16.1) [0, 23.4]	87.7% (85.0%–90.5%)	85.1% (82.2%–88.1%)	84.2% (81.1%–87.3%)
Diagnoses with a primary diagnosis that can be either managed by single‐ventricle or biventricular pathway					
TGA	Total (TGA)	11.1 (5.4–16.7) [0, 23.4]	94.4% (93.7%–95.1%)	93.5% (92.7%–94.3%)	93.1% (92.2%–93.9%)
	Complex TGA and PS	11.2 (6.0–17.0) [0, 23.4]	93.0% (90.7%–95.4%)	90.5% (87.8%–93.2%)	89.8% (87.0%–92.7%)
	Complex TGA without PS	10.3 (4.9–15.5) [0, 23.3]	92.2% (90.7%–93.6%)	91.1% (89.6%–92.7%)	90.3% (88.7%–92.0%)
	TGA with intact ventricular septum	11.7 (5.7–17.3) [0, 23.4]	96.1% (95.3%–96.9%)	95.7% (94.8%–96.6%)	95.5% (94.6%–96.4%)
PA	Total (PA)	9.5 (3.2–16.1) [0, 23.4]	86.5% (84.9%–88.2%)	82.1% (80.3%–84.0%)	81.0% (79.1%–82.9%)
	PA and VSD	9.9 (3.7–16.0) [0, 23.4]	89.2% (87.4%–91.0%)	83.4% (81.2%–85.6%)	82.1% (79.8%–84.4%)
	PA with intact ventricular septum	8.9 (2.0–16.6) [0, 23.3]	80.6% (77.2%–84.1%)	79.3% (75.9%–82.9%)	78.6% (75.1%–82.2%)
AVSD	Total (AVSD)	11.0 (5.2–16.7) [0, 23.4]	92.3% (91.5%–93.1%)	89.2% (88.2%–90.1%)	88.5% (87.5%–89.5%)
	Tetralogy AVSD	9.3 (4.0–15.2) [0.1, 23.3]	91.1% (87.4%–95.0%)	83.3% (78.4%–88.5%)	80.8% (75.5%–86.5%)
	Unbalanced AVSD	5.9 (0.9–13.1) [0, 23.3]	74.2% (69.3%–79.4%)	64.3% (59.0%–70.1%)	62.5% (57.1%–68.4%)
	Partial AVSD	13.2 (8.4–18.2) [0, 23.4]	98.5% (97.7%–99.2%)	97.0% (96.0%–98.0%)	96.7% (95.6%–97.7%)
	Complete AVSD	10.5 (4.7–16.4) [0, 23.4]	91.8% (90.7%–92.8%)	89.1% (87.9%–90.3%)	88.5% (87.3%–89.7%)
Diagnoses that are exclusively biventricular					
TOF	Total (TOF)	11.4 (6.3–16.8) [0, 23.4]	97.3% (96.8%–97.8%)	95.7% (95.2%–96.3%)	95.1% (94.5%–95.8%)
	Tetralogy with absent pulmonary valve	12.1 (7.7–17.6) [0, 23.3]	94.6% (91.3%–97.9%)	92.9% (89.2%–96.7%)	91.6% (87.5%–95.8%)
	Tetralogy with DORV	9.3 (5.1–14.1) [0, 23.2]	95.6% (93.8%–97.3%)	91.5% (89.2%–93.9%)	90.5% (88.0%–93.1%)
	Standard tetralogy	11.7 (6.4–17.1) [0, 23.4]	97.7% (97.2%–98.1%)	96.5% (95.9%–97.0%)	95.9% (95.3%–96.6%)
AS	Total (AS)	13.0 (7.3–18.2) [0, 23.4]	95.5% (94.5%–96.5%)	94.7% (93.7%–95.8%)	94.4% (93.3%–95.6%)
	AS and mutilevel left‐sided heart obstruction	12.2 (6.7–17.2) [0, 23.3]	94.7% (92.6%–96.8%)	93.0% (90.6%–95.5%)	92.1% (89.5%–94.7%)
	Isolated AS	13.2 (7.6–18.3) [0, 23.4]	95.8% (94.7%–96.9%)	95.4% (94.2%–96.6%)	95.3% (94.1%–96.5%)
Coarctation	Total (coarctation)	11.6 (6.1–17.4) [0, 23.4]	97.6% (97.2%–98.1%)	96.9% (96.4%–97.4%)	96.7% (96.2%–97.3%)
	Coarctation plus VSD	9.9 (5.1–15.5) [0, 23.4]	96.6% (95.6%–97.6%)	95.6% (94.5%–96.7%)	95.6% (94.5%–96.7%)
	Isolated coarctation	12.3 (6.8–18.4) [0, 23.4]	98.1% (97.6%–98.6%)	97.5% (96.9%–98.1%)	97.3% (96.7%–97.9%)
VSD	Total (VSD)	11.4 (6.2–16.7) [0, 23.4]	98.4% (98.1%–98.7%)	97.3% (96.9%–97.7%)	96.9% (96.4%–97.3%)
	Multiple VSDs	11.0 (5.3–16.4) [0, 23.2]	96.4% (94.7%–98.2%)	93.7% (91.4%–96.1%)	92.3% (89.7%–95.0%)
	Isolated VSD	11.4 (6.4–16.8) [0, 23.4]	98.5% (98.2%–98.8%)	97.6% (97.2%–98.0%)	97.2% (96.8%–97.6%)

Survival rates (Kaplan–Meier) with 95% CIs at 1, 5, and 10 years old. The cohort was based on on National Congenital Heart Diseases Audit data from April 2000 to March 2022. AS indicates aortic stenosis; AVSD, atrioventricular septal defect; CHD, congenital heart disease; DORV, double‐outlet right ventricle; FUH, functionally univentricular heart; HLHS, hypoplastic left heart syndrome; IQR, interquartile range; PA, pulmonary atresia; PS, pulmonary stenosis; TGA, transposition of the great arteries; TOF, tetralogy of Fallot; and VSD, ventricular septal defect.

There were differences in survival rates between CHD subgroups, which we show individually in Table 2, the most pronounced of which was for AVSD (10‐year survival rates of 80.8% for tetralogy with AVSD, 62.5% for unbalanced AVSD, 96.7% for partial AVSD, and 88.5% for complete AVSD).

The reintervention metrics for each CHD diagnosis are given in Table 4 and can be summarized as reintervention cumulative incidence at 10 years old: HLHS: 54.5% (95% CI, 51.5%–57.3%), FUH: 57.3% (95% CI, 53.9%–60.5%), TGA: 20.9% (95% CI, 19.5%–22.3%), PA: 66.8% (95% CI, 64.2%–69.1%), AVSD: 21.6% (95% CI, 20.3%–23.0%), TOF: 26.6% (95% CI, 25.2%–28.0%), AS: 31.2% (95% CI, 28.8%–33.6%), coarctation: 19.8% (95% CI, 18.6%–21.1%), and VSD: 6.1% (95% CI, 5.5%–6.8%). There were differences in reintervention occurrence by CHD subgroup, the most pronounced for TGA (10‐year reintervention of 59.3% for complex TGA with PS, 25.4% for complex TGA without PS, and 9.8% for TGA with intact ventricular septum). Of note, patients with suspected missing data (305 [1.0%]) were excluded from the calculations of reintervention metrics.

**Table 4:** Reintervention Metrics in Each CHD Diagnosis Group

Diagnosis	Diagnosis subgroup	Follow‐up time, y	Cumulative incidence of reintervention (any type)		
		Median (IQR) [minimum, maximum]	At age 1 y	At age 5 y	At age 10 y
Diagnoses that are exclusively functionally single ventricle					
HLHS	HLHS	0.3 (0.1–3.5) [0, 21.7]	37.4% (34.7%–40.1%)	48.7% (45.8%–51.5%)	54.5% (51.5%–57.3%)
FUH	Total (FUH)	3.2 (0.3–8.6) [0, 22.0]	27.9% (25.2%–30.8%)	46.0% (42.8%–49.2%)	57.3% (53.9%–60.5%)
	Double‐inlet ventricle	3.9 (0.5–9.0) [0, 21.6]	26.5% (22.5%–30.8%)	46.0% (41.1%–50.8%)	58.7% (53.5%–63.5%)
	Tricuspid atresia	2.7 (0.3–8.0) [0, 22.0]	29.1% (25.4%–33.0%)	46.3% (41.9%–50.5%)	56.2% (51.6%–60.5%)
Diagnoses with a primary diagnosis that can be either managed by functionally single‐ventricle or biventricular pathway					
TGA	Total (TGA)	7.1 (1.3–13.4) [0, 22.0]	13.2% (12.1%–14.3%)	18.4% (17.1%–19.7%)	20.9% (19.5%–22.3%)
	Complex TGA and PS	2.4 (0.4–8.6) [0, 21.9]	30.3% (25.9%–34.7%)	50.3% (45.3%–55.1%)	59.3% (54.0%–64.2%)
	Complex TGA without PS	6.1 (0.6–12.1) [0, 21.9]	17.2% (15.2%–19.4%)	22.0% (19.6%–24.4%)	25.4% (22.8%–28.0%)
	TGA with intact ventricular septum	8.9 (2.7–14.7) [0, 22.0]	7.0% (6.0%–8.2%)	9.3% (8.1%–10.7%)	9.8% (8.6%–11.2%)
PA	Total (PA)	1.1 (0.2–4.4) [0, 21.9]	36.0% (33.6%–38.3%)	59.3% (56.8%–61.7%)	66.8% (64.2%–69.1%)
	PA and VSD	1.3 (0.5–4.2) [0, 21.9]	32.0% (29.3%–34.8%)	62.0% (59.0%–64.8%)	69.9% (67.0%–72.7%)
	PA with intact ventricular septum	0.4 (0.0–5.3) [0, 21.9]	44.9% (40.5%–49.2%)	52.8% (48.2%–57.1%)	58.9% (54.2%–63.3%)
AVSD	Total (AVSD)	7.4 (2.0–13.7) [0, 22.0]	9.5% (8.7%–10.4%)	17.6% (16.4%–18.8%)	21.6% (20.3%–23.0%)
	Tetralogy with AVSD	3.8 (1.0–10.3) [0, 22.0]	18.2% (13.2%–23.8%)	34.3% (27.6%–41.0%)	39.1% (32.0%–46.2%)
	Unbalanced AVSD	1.3 (0.3–5.8) [0, 21.8]	29.4% (24.1%–34.9%)	41.8% (35.8%–47.7%)	49.0% (42.5%–55.2%)
	Partial AVSD	10.3 (5.1–15.8) [0, 22.0]	4.1% (3.0%–5.3%)	11.8% (10.0%–13.9%)	16.5% (14.3%–18.9%)
	Complete AVSD	7.0 (1.7–13.5) [0, 22.0]	9.2% (8.1%–10.3%)	16.1% (14.7%–17.6%)	19.6% (18.0%–21.3%)
Diagnoses that are exclusively biventricular					
TOF	Total (TOF)	7.2 (2.3–13.0) [0, 22.0]	8.8% (8.1%–9.7%)	21.1% (19.9%–22.3%)	26.6% (25.2%–28.0%)
	Tetralogy with absent pulmonary valve	6.2 (1.6–11.7) [0, 21.5]	13.2% (8.8%–18.6%)	35.3% (28.3%–42.4%)	47.5% (39.4%–55.2%)
	Tetralogy with DORV	4.6 (1.2–9.7) [0, 20.6]	15.8% (12.8%–19.1%)	33.9% (29.7%–38.1%)	39.6% (35.0%–44.2%)
	Standard tetralogy	7.7 (2.6–13.4) [0, 22.0]	7.7% (6.9%–8.6%)	18.6% (17.4%–19.9%)	23.8% (22.3%–25.2%)
AS	Total (AS)	8.5 (2.2–14.1) [0, 22.0]	13.4% (11.8%–15.2%)	21.9% (19.9%–24.0%)	31.2% (28.8%–33.6%)
	AS and multilevel left‐sided heart obstruction	4.7 (0.5–11.4) [0, 21.9]	26.6% (22.6%–30.9%)	40.9% (36.1%–45.5%)	52.3% (47.1%–57.2%)
	Isolated AS	9.5 (3.5–14.9) [0, 22.0]	8.7% (7.2%–10.4%)	14.9% (12.9%–17.1%)	23.4% (20.9%–26.1%)
Coarctation	Total (coarctation)	8.1 (2.1–14.3) [0, 22.0]	11.8% (10.8%–12.8%)	16.8% (15.7%–18.0%)	19.8% (18.6%–21.1%)
	Coarctation plus VSD	5.0 (0.9–11.7) [0, 22.0]	18.0% (15.9%–20.1%)	25.9% (23.5%–28.4%)	27.9% (25.3%–30.5%)
	Isolated coarctation	9.4 (3.0–15.2) [0, 22.0]	9.0% (8.0%–10.1%)	12.8% (11.6%–14.1%)	16.2% (14.8%–17.7%)
VSD	Total (VSD)	9.4 (4.2–15.0) [0, 22.0]	2.8% (2.4%–3.2%)	5.3% (4.8%–5.9%)	6.1% (5.5%–6.8%)
	Multiple VSDs	6.3 (2.2–13.6) [0, 21.9]	8.8% (6.3%–11.8%)	21.1% (17.2%–25.3%)	22.2% (18.1%–26.5%)
	Isolated VSD	9.5 (4.5–15.0) [0, 22.0]	2.3% (2.0%–2.7%)	4.2% (3.7%–4.8%)	5.0% (4.4%–5.6%)

Reintervention cumulative incidence with 95% CIs at 1, 5, and 10 years old, taking account of death and heart transplant without reintervention as competing events. The follow‐up period considered the occurrence of the reintervention or competing events (death and heart transplant), whichever came earlier, as end points. Patients with suspected missing or miscoded data (305 [1%]) were removed from the reintervention monitoring. Reintervention metrics by types (surgical and interventional cardiology) are presented in the supplemental material. Cohort based on National Congenital Heart Diseases Audit data from April 2000 to March 2022. AS indicates aortic stenosis; AVSD, atrioventricular septal defect; CHD, congenital heart disease; DORV, double‐outlet right ventricle; FUH, functionally univentricular heart; HLHS, hypoplastic left heart syndrome; IQR, interquartile range; PA, pulmonary atresia; PS, pulmonary stenosis; TGA, transposition of the great arteries; TOF, tetralogy of Fallot; and VSD, ventricular septal defect.

The Kaplan–Meier curves and reintervention cumulative incidence function charts for each sentinel CHD are shown by subgroups in Figures S2 and S3. We present a breakdown of the captured reintervention numbers by type of reintervention (bypass surgery, cardiac surgery nonbypass, hybrid procedures, interventional catheters, and electrophysiology types) for each sentinel CHD in Table S1 and the cumulative incidence of reintervention by surgery and interventional cardiology types in Tables S2 and S3.

## DISCUSSION

### Summary of Findings

As the 30‐day mortality rate for pediatric cardiac surgery has consistently fallen below 2% in England, service providers, commissioners, and patients and their families support a wider range of outcomes being monitored routinely by national audit. We combined clinical expertise, patient and family views, and statistical analysis of the national audit database, to select 9 sentinel CHD diagnoses and a set of longer‐term outcomes (1‐, 5‐, and 10‐year survival and reintervention rates) suitable for routine monitoring. To support the feasibility of future routine monitoring, we also defined treatment pathways for each diagnosis and developed algorithms to assign the applicable sentinel CHD types and treatment pathways to individual patients in the data. The presented outcomes are of interest to all stakeholders because they provide a more complete picture than 30‐day metrics. This is because for these CHDs, the treatment pathway often involves more than one surgery; a range of additional risk factors may evolve over time;^29^, ^31^, ^32^ and there is the possibility of late postdischarge death. Moreover, these outcomes reflect contemporary treatments in a growing population of survivors, and such information is sparse.^33^

Our 9 sentinel CHDs captured nearly half of all operations undertaken nationally and a range of severities, which is advantageous for metrics for assessing overall care quality. Severity is illustrated by the 10‐year survival rates, which ranged from 57.6% (95% CI, 54.9%–60.4%) for children with HLHS to 97.3% (95% CI, 96.7%–97.9%) for children with isolated VSD. Reinterventions have rarely been reported as metrics, given the complexities in ascertaining these outcomes. However, our work has shown that it is feasible and demonstrates their importance, given that by the age of 10 years, these occurred in more than half of children with HLHS, FUH, and PA and approximately 20% to 50% of children with the other CHD diagnoses.

### Context

Most quality assurance registries and audits currently focus their reporting on short‐term in‐hospital outcomes of mortality and postoperative complications.^2^, ^34^, ^35^ Both of these were raised as important metrics within the patient online discussion forums undertaken in the United Kingdom^11^ and within the recent James Lind Alliance Priortiy Setting Partnership for CHD,^36^ and are reported by NCHDA. A few national registries have already reported long‐term outcomes but they are more limited in scope than our analysis. For example, the Swedcon registry in Sweden reports aggregated quality‐of‐life data:^32^ patient‐reported outcomes including quality of life, which were also prioritized by patients and families in the United Kingdom.^11^, ^36^ The Australia and New Zealand Fontan Registry reports long‐term survival in a range of publications but captures children only after they reach the third stage of single‐ventricle palliation and only for a restricted set of diagnoses.^31^ Outside of the context of routine monitoring, population‐based data on long‐term outcomes with CHD are sparse: a systematic review and metanalysis of population‐based studies of long‐term survival with CHD published in 2016 found only 16 worldwide and calculated a pooled 10‐year survival rate of 81.4% (95% CI, 73.8–87.9).^33^ This systematic review included population‐based data from 3 sites in Arizona, Arkansas, and Atlanta, Georgia, within the Centers for Disease Control and Prevention–funded CH STRONG study.^37^ This study importantly highlighted the role of disparities in determining longer‐term survival for individuals with CHD.^38^

### Strengths and Limitations

Although we selected for inclusion 9 sentinel CHDs with significant subtypes to capture a wide range of key conditions affecting young children, certain rarer CHDs that are nonetheless important (as shown in Table 1) were not included. Our registry‐based study reflects practice in England and Wales and is limited by data quality; however, cardiac codes in NCHDA were of high quality over the entire study period. There is no perfect method for categorizing CHD diagnoses given the heterogeneity of CHD; however, we ensured robust definitions through the involvement of experienced clinicians in defining CHD diagnoses and data quality thresholds. In addition, NCHDA is a procedure‐based data set, so patients who did not undergo any surgical or interventional cardiac procedures were not incorporated into the study. Children with CHD may be affected by conditions that influence their outcomes such as low weight, preterm birth, and noncardiac comorbidities.^15^ Although we report the rates of these conditions, their influence on the outcomes was not considered in this descriptive study. This current study provides methods of analysis for longer‐term outcomes by sentinel CHD diagnoses but did not take the next important steps required to use them to explore practice and evaluate quality of care.

We note that the following metrics were highlighted as important by patients and families in the online forums^11^ but are not measurable within the current routinely collected registry data sets: use of medications, pregnancy risks and outcomes, availability of support services, cancellations of surgery, and delayed follow‐up. A limitation of online forums as a method to gather patient and family views is that these may capture the views of people who have access to the internet, excluding those who do not, which might undermine diversity of representation.

### Future Directions

Although we have demonstrated that it is feasible to use routine NHS data sets to evaluate important longer‐term outcome metrics for CHD in childhood, there are residual challenges before these could be reported by the national audit. Difficulties relate to the important variation in outcomes by CHD subgroups, the small number of children starting treatment for each CHD and subgroup within individual centers, and how to address these issues when assessing center‐level outcomes. Therefore, reporting of these CHD outcome metrics at the national level inclusive of subgroup outcomes might be the best place to start. Future uses of our methods of analysis could include the evaluation of health care costs over the longer‐term and any influences of social factors on outcomes. Patients/families identified a range of longer‐term outcome metrics not captured by national audit, emphasizing the need to explore and evaluate a wider range of more complex outcome metrics in the future.

## Sources of Funding

This study was funded by the National Institute for Health and Care Research (NIHR) Department of Health and Social Care Policy Research Programme (grant number PR‐R20‐0318‐23 001). The views expressed are those of the authors and not necessarily those of the NIHR or the Department of Health and Social Care. Katherine Brown and Jo Wray were supported by Great Ormond Street Hospital Biomedical Research Centre.

## Disclosures

None.

## Supporting information

Tables S1–S3
Figures S1–S3
