# Supplementary material for: Evaluating Long‐Term Outcomes of Children Undergoing Surgical Treatment for Congenital Heart Disease for National Audit in England and Wales
Source: J Am Heart Assoc. 2024 Oct 29;13(21):e035166. doi: 10.1161/JAHA.124.035166 (PMC11935704; doi:10.1161/JAHA.124.035166)
Supplement: Supplementary file 1 — Tables S1–S3 Figures S1–S3 [file JAH3-13-e035166-s001.pdf]

Supplemental Material

Table S1: Breakdown of reintervention types in each congenital heart disease diagnosis.

Patients with suspected missing or miss coded data, 305 (1%), have been removed from the reintervention analysis. Non-bypass surgical reinterventions include pacemakers/ pacemaker related procedures.

AVSD=atrioventricular septal defect; DORV=double outlet right ventricle; EP=electrophysiology intervention; FUH=functionally univentricular heart; HLHS=hypoplastic left heart syndrome; PS=pulmonary stenosis; TGA= transposition of the great arteries; TOF=tetralogy of Fallot; VSD=ventricular septal defect.

**Diagnosis**	**Diagnosis subgroup**	**Surgical reintervention**				**Interventional cardiology reintervention**		
		**Number (total)**	**Number of bypass operations (%)**	**Number of non-bypass operations (%)**	**Number of hybrid procedures (%)**	**Number (total)**	**Number of interventional catheters (%)**	**Number of EP procedures (%)**
**Diagnoses that are exclusively single ventricle**								
**HLHS**	HLHS	384	257 (66.9%)	118 (30.7%)	9 (2.3%)	1,091	1,067 (97.8%)	24 (2.2%)
**FUH**	Total (FUH)	389	185 (47.6%)	199 (51.2%)	5 (1.3%)	718	699 (97.4%)	19 (2.7%)
	Double inlet ventricle	175	81 (46.3%)	91 (52.0%)	3 (1.7%)	354	340 (96.0%)	14 (4.0%)
	Tricuspid atresia	214	104 (48.6%)	108 (50.5%)	2 (0.9%)	364	359 (98.6%)	5 (1.4%)
**Diagnoses with primary diagnosis that can be either managed by single ventricle or biventricular pathway**								
**TGA**	Total (TGA)	665	468 (70.4%)	185 (27.8%)	12 (1.8%)	747	700 (93.7%)	47 (6.3%)
	Complex TGA & pulmonary stenosis (PS)	278	191 (68.7%)	83 (29.9%)	4 (1.4%)	300	279 (93.0%)	21 (7.0%)
	Complex TGA without PS	271	173 (63.9%)	92 (34.0%)	6 (2.2%)	230	218 (94.8%)	12 (5.2%)
	TGA with intact ventricular septum	116	104 (89.7%)	10 (8.6%)	2 (1.7%)	217	203 (93.4%)	14 (6.5%)
	Total (Pulmonary atresia)	1,212	927 (76.5%)	271 (22.4%)	14 (1.2%)	1,599	1,566 (97.9%)	33 (2.1%)

**Pulmonary atresia**	Pulmonary atresia & VSD	986	787 (79.8%)	186 (18.9%)	13 (1.3%)	1,257	1,226 (97.5%)	31 (2.5%)
	Pulmonary atresia with intact ventricular septum	226	140 (61.9%)	85 (37.6%)	1 (0.4%)	342	340 (99.4%)	2 (0.6%)
**AVSD**	Total (AVSD)	1,180	873 (74.0%)	302 (25.6%)	5 (0.4%)	337	231 (68.6%)	106 (31.5%)
	Tetralogy AVSD	91	63 (69.2%)	28 (30.8%)	none	80	74 (92.5%)	6 (7.5%)
	Unbalanced AVSD	135	80 (59.3%)	54 (40.0%)	1 (0.7%)	98	95 (96.9%)	3 (3.1%)
	Partial AVSD	271	221 (81.5%)	49 (18.1%)	1 (0.4%)	39	14 (35.9%)	25 (64.1%)
	Complete AVSD	683	509 (74.5%)	171 (25.0%)	3 (0.4%)	120	48 (40.0%)	72 (60.0%)
**Diagnoses that exclusively biventricular**								
**Tetralogy of Fallot (TOF)**	Total (TOF)	1,170	982 (83.9%)	170 (14.5%)	18 (1.5%)	1,177	1,106 (94.0%)	71 (6.0%)
	Tetralogy absent pulmonary valve	102	96 (94.1%)	6 (5.9%)	none	87	85 (97.7%)	2 (2.3%)
	Tetralogy with DORV	187	146 (78.1%)	37 (19.8%)	4 (2.1%)	181	173 (95.6%)	8 (4.2%)
	Standard tetralogy	881	740 (84.0%)	127 (14.4%)	14 (1.6%)	909	848 (93.3%)	61 (6.7%)
**Aortic stenosis**	Total (Aortic stenosis)	656	616 (93.9%)	35 (5.3%)	5 (0.8%)	283	260 (91.9%)	23 (8.1%)
	Aortic stenosis & muti-level left heart obstruction	306	272 (88.9%)	32 (10.5%)	2 (0.7%)	138	122 (88.4%)	16 (11.6%)
	Isolated aortic stenosis	350	344 (98.2%)	3 (0.9%)	3 (0.9%)	145	138 (95.2%)	7 (4.8%)
**Coarctation**	Total (Coarctation)	572	382 (66.8%)	189 (33.0%)	1 (0.2%)	591	572 (96.8%)	19 (3.2%)
	Coarctation plus VSD	317	195 (61.5%)	122 (38.5%)	none	182	170 (93.4%)	12 (6.6%)
	Isolated coarctation	255	187 (73.3%)	67 (26.3%)	1 (0.4%)	409	402 (98.3%)	7 (1.7%)
**VSD**	Total (VSD)	389	168 (43.2%)	168 (43.2%)	217 (55.8%)	168	89 (53.0%)	79 (47.0%)

	Multiple VSD	94	41 (43.6%)	52 (55.3%)	1 (1.1%)	34	26 (76.5%)	8 (23.5%)
	Isolated VSD	295	127 (43.1%)	165 (55.9%)	3 (1.0%)	134	63 (47.0%)	71 (53.0%)

Table S2. Surgical reintervention cumulative incidence in each congenital heart disease diagnosis for LTO monitoring.

Surgical reintervention cumulative incidence with 95% confidence interval at 1 year, 5 years and 10 years old, taking account of death and heart transplant without surgical reintervention as competing events. The follow-up period considered the occurrence of the reintervention or competing events (death and heart transplant), whichever came earlier, as endpoints. Patients with suspected missing or miss coded data, 305 (1%), have been removed from the reintervention monitoring.

AVSD=atrioventricular septal defect; DORV=double outlet right ventricle; FUH=functionally univentricular heart; HLHS=hypoplastic left heart syndrome; PS=pulmonary stenosis; TGA= transposition of the great arteries; TOF=tetralogy of Fallot; VSD=ventricular septal defect.

Diagnosis	Diagnosis subgroup	Follow-up time (years) median (IQR) [min, max]	Cumulative incidence of surgical reintervention		
			at age 1 year	at age 5 years	at age 10 years
**Diagnoses that are exclusively functionally single ventricle**					
**HLHS**	HLHS	1.1 (0.1-8.9) [0, 22.0]	16.9% (14.8%-19.0%)	22.0% (19.6%-24.4%)	23.9% (21.4%-26.4%)
**FUH**	Total (FUH)	5.7 (0.9-12.5) [0, 22.0]	17.3% (15.0%-19.8%)	25.0% (22.3%-27.8%)	28.6% (25.7%-31.7%)
	Double inlet ventricle	6.5 (1.7-12.4) [0, 21.9]	14.9% (11.7%-18.4%)	23.1% (19.2%-27.3%)	26.7% (22.4%-31.2%)
	Tricuspid atresia	5.0 (0.6-12.4) [0, 22.0]	19.4% (16.2%-22.8%)	26.6% (22.9%-30.4%)	30.3% (26.3%-34.4%)
**Diagnoses with primary diagnosis that can be either managed by functionally single ventricle or biventricular pathway**					
**TGA**	Total (TGA)	8.4 (2.2-14.0) [0, 22.0]	7.6% (6.8%-8.5%)	11.1% (10.1%-12.2%)	13.1% (12.0%-14.3%)
	Complex TGA & pulmonary stenosis (PS)	4.9 (0.9-10.6) [0, 21.9]	19.6% (15.9%-23.5%)	34.7% (30.1%-39.4%)	42.5% (37.4%-47.6%)
	Complex TGA without pulmonary stenosis	7.4 (1.3-12.9) [0, 21.9]	10.9% (9.2%-12.7%)	14.2% (12.3%-16.3%)	16.2% (14.1%-18.5%)
	TGA with intact ventricular septum	9.5 (3.6-15.3) [0, 22.0]	3.1% (2.4%-3.9%)	4.2% (3.4%-5.1%)	4.9% (3.9%-5.9%)
**Pulmonary atresia**	Total (Pulmonary atresia)	2.1 (0.5-8.3) [0, 21.9]	21.7% (19.7%-23.8%)	41.0% (38.5%-43.4%)	49.2% (46.5%-51.8%)

	Pulmonary atresia & VSD	2.1 (0.7-7.4) [0, 21.9]	21.3% (19.0%-23.8%)	46.6% (43.5%-49.6%)	56.4% (53.1%-59.5%)
	Pulmonary atresia with intact ventricular septum	2.7 (0.1-10.8) [0, 21.9]	22.5% (19.0%-26.3%)	27.8% (23.9%-31.9%)	32.2% (27.8%-36.5%)
**AVSD**	Total (AVSD)	7.7 (2.2-14.0) [0, 22.0]	8.2% (7.3%-9.0%)	15.6% (14.5%-16.7%)	19.1% (17.9%-20.4%)
	Tetralogy with AVSD	4.4 (1.4-10.7) [0, 22.0]	11.3% (7.4%-16.1%)	26.0% (20.0%-32.5%)	29.5% (23.0%-36.3%)
	Unbalanced AVSD	2.0 (0.4-8.4) [0, 21.8]	20.6% (16.0%-25.6%)	30.2% (24.7%-35.8%)	33.9% (28.0%-39.8%)
	Partial AVSD	10.4 (5.4-15.9) [0, 22.0]	3.6% (2.6%-4.8%)	11.2% (9.4%-13.2%)	15.7% (13.4%-18.0%)
	Complete AVSD	7.1 (1.8-13.5) [0, 22.0]	8.5% (7.5%-9.6%)	15.1% (13.7%-16.5%)	18.2% (16.7%-19.9%)
**Diagnoses that are exclusively biventricular**					
**Tetralogy of Fallot (TOF)**	Total (TOF)	8.1 (3.1-13.5) [0, 22.0]	5.8% (5.1%-6.5%)	13.5% (12.5%-14.5%)	18.1% (16.9%-19.4%)
	Tetralogy absent pulmonary valve	7.7 (2.2-12.6) [0, 22.0]	8.3% (4.8%-12.8%)	26.0% (19.7%-32.8%)	37.8% (30.0%-45.5%)
	Tetralogy with DORV	6.1 (1.9-11.0) [0, 21.5]	9.7% (7.3%-12.4%)	20.5% (17.0%-24.2%)	26.7% (22.5%-31.1%)
	Standard tetralogy	8.5 (3.3-13.8) [0, 22.0]	5.2% (4.5%-5.9%)	11.9% (10.9%-13.0%)	16.0% (14.8%-17.3%)
**Aortic Stenosis**	Total (Aortic Stenosis)	9.2 (3.2-14.7) [0, 22.0]	8.4% (7.1%-9.8%)	16.6% (14.8%-18.5%)	25.1% (22.9%-27.4%)
	Aortic stenosis & multi-level left heart obstruction	5.9 (1.2-12.5) [0, 21.9]	18.8% (15.2%-22.6%)	33.8% (29.2%-38.3%)	45.5% (40.4%-50.5%)
	Isolated aortic stenosis	10.0 (4.1-15.2) [0, 22.0]	4.7% (3.6%-6.1%)	10.3% (8.6%-12.2%)	17.6% (15.3%-20.1%)
**Coarctation**	Total (Coarctation)	9.1 (3.1-15.2) [0, 22.0]	5.8% (5.1%-6.5%)	10.3% (9.4%-11.2%)	11.8% (10.8%-12.8%)

	Coarctation plus VSD	6.1 (1.7-12.6) [0, 22.0]	10.9% (9.3%-12.7%)	19.1% (17.0%-21.4%)	20.2% (18.0%-22.6%)
	Isolated coarctation	10.2 (4.2-16.5) [0, 22.0]	3.5% (2.9%-4.2%)	6.4% (5.5%-7.3%)	8.1% (7.1%-9.2%)
**VSD**	Total (VSD)	9.5 (4.4-15.0) [0, 22.0]	2.5% (2.2%-3.0%)	4.2% (3.7%-4.7%)	4.8% (4.2%-5.3%)
	Multiple VSDs	7.3 (2.4-13.9) [0, 21.9]	8.1% (5.7%-11.0%)	16.7% (13.2%-20.6%)	18.1% (14.4%-22.2%)
	Isolated VSD	9.7 (4.5-15.1) [0, 22.0]	2.2% (1.8%-2.6%)	3.4% (2.9%-3.8%)	3.8% (3.4%-4.4%)

Table S3. Interventional cardiology reintervention cumulative incidence in each congenital heart disease diagnosis for LTO monitoring.

Interventional cardiology reintervention cumulative incidence with 95% confidence interval at 1 year, 5 years and 10 years old, taking account of death and heart transplant without reintervention as competing events. The follow-up period considered the occurrence of the reintervention or competing events (death and heart transplant), whichever came earlier, as endpoints. Patients with suspected missing or miss coded data, 305 (1%), have been removed from the reintervention monitoring.

AVSD=atrioventricular septal defect; DORV=double outlet right ventricle; FUH=functionally univentricular heart; HLHS=hypoplastic left heart syndrome; PS=pulmonary stenosis; TGA= transposition of the great arteries; TOF=tetralogy of Fallot; VSD=ventricular septal defect.

Diagnosis	Diagnosis subgroup	Follow-up time (years) median (IQR) [min, max]	Cumulative incidence of interventional cardiology reintervention		
			at age 1 year	at age 5 years	at age 10 years
**Diagnoses that are exclusively functionally single ventricle**					
**HLHS**	HLHS	0.4 (0.1-4.1) [0, 21.7]	28.6% (26.1%-31.2%)	39.8% (36.9%-42.5%)	45.7% (42.8%-48.6%)
**FUH**	Total (FUH)	4.5 (0.9-10.5) [0, 22.0]	15.7% (13.5%-18.1%)	32.7% (29.7%-35.8%)	44.7% (41.2%-48.0%)
	Double inlet ventricle	4.6 (1.1-10.3) [0, 22.0]	16.7% (13.4%-20.4%)	35.1% (30.5%-39.8%)	49.2% (44.0%-54.2%)
	Tricuspid atresia	4.3 (0.6-10.7) [0, 22.0]	14.9% (12.1%-18.0%)	31.0% (27.0%-35.0%)	41.1% (36.6%-45.6%)
**Diagnoses with primary diagnosis that can be either managed by functionally single ventricle or biventricular pathway**					
**TGA**	Total (TGA)	8.1 (2.1-14.0) [0, 22.0]	7.6% (6.8%-8.5%)	11.4% (10.3%-12.5%)	13.5% (12.3%-14.7%)
	Complex TGA & pulmonary stenosis (PS)	5.0 (1.0-11.2) [0, 21.9]	17.0% (13.6%-20.8%)	31.3% (26.8%-35.9%)	39.3% (34.2%-44.4%)
	Complex TGA without pulmonary stenosis	7.2 (1.5-12.8) [0, 21.9]	9.3% (7.7%-11.0%)	12.4% (10.6%-14.3%)	15.1% (13.0%-17.3%)
	TGA with intact ventricular septum	9.1 (3.1-15.0) [0, 22.0]	4.6% (3.8%-5.6%)	6.5% (5.4%-7.7%)	6.9% (5.8%-8.1%)
**Pulmonary atresia**	Total (Pulmonary atresia)	2.1 (0.5-8.5) [0, 22.0]	22.1% (20.1%-24.2%)	41.9% (39.4%-44.3%)	48.2% (45.6%-50.8%)

	Pulmonary atresia & VSD	2.5 (0.8-9.1) [0, 22.0]	18.2% (15.9%-20.5%)	42.4% (39.4%-45.4%)	49.3% (46.2%-52.4%)
	Pulmonary atresia with intact ventricular septum	0.9 (0.1-7.5) [0, 21.9]	31.0% (27.0%-35.1%)	40.5% (36.1%-44.9%)	45.5% (40.9%-50.0%)
**AVSD**	Total (AVSD)	9.3 (3.5-15.0) [0, 22.0]	1.9% (1.5%-2.3%)	3.6% (3.0%-4.2%)	5.0% (4.3%-5.8%)
	Tetralogy with AVSD	6.8 (1.6-12.3) [0.1, 22.0]	8.9% (5.4%-13.3%)	15.7% (11.0%-21.2%)	20.7% (14.9%-27.2%)
	Unbalanced AVSD	2.4 (0.5-8.6) [0, 22.0]	11.8% (8.3%-16.0%)	18.7% (14.2%-23.7%)	25.8% (20.3%-31.7%)
	Partial AVSD	11.6 (6.7-16.6) [0, 22.0]	0.7% (0.3%-1.4%)	1.4% (0.8%-2.3%)	2.0% (1.2%-3.0%)
	Complete AVSD	8.9 (3.2-14.8) [0, 22.0]	0.8% (0.5%-1.2%)	2.0% (1.5%-2.6%)	3.1% (2.4%-3.9%)
**Diagnoses that are exclusively biventricular**					
**Tetralogy of Fallot (TOF)**	Total (TOF)	8.5 (3.2-14.1) [0, 22.0]	4.2% (3.6%-4.8%)	12.2% (11.2%-13.2%)	15.5% (14.4%-16.7%)
	Tetralogy absent pulmonary valve	8.0 (2.1-14.3) [0, 21.5]	7.2% (4.0%-11.6%)	20.6% (14.9%-26.9%)	25.1% (18.7%-32.0%)
	Tetralogy with DORV	6.0 (1.9-11.4) [0, 21.9]	7.1% (5.1%-9.6%)	19.5% (16.1%-23.2%)	22.9% (19.1%-26.9%)
	Standard tetralogy	8.8 (3.5-14.5) [0, 22.0]	3.6% (3.0%-4.2%)	10.8% (9.8%-11.9%)	14.0% (12.9%-15.3%)
**Aortic Stenosis**	Total (Aortic Stenosis)	10.3 (3.9-15.8) [0, 22.0]	7.6% (6.3%-8.9%)	10.5% (9.0%-12.1%)	13.6% (11.8%-15.4%)
	Aortic stenosis & multi-level left heart obstruction	8.5 (1.9-14.3) [0, 21.9]	14.4% (11.2%-17.9%)	18.7% (15.1%-22.6%)	23.6% (19.5%-28.0%)
	Isolated aortic stenosis	10.9 (4.8-16.5) [0, 22.0]	5.1% (3.9%-6.4%)	7.5% (6.1%-9.1%)	9.9% (8.2%-11.8%)
**Coarctation**	Total (Coarctation)	9.2 (3.2-14.8) [0, 22.0]	7.3% (6.5%-8.1%)	8.6% (7.8%-9.5%)	10.4% (9.5%-11.4%)
	Coarctation plus VSD	7.2 (2.4-13.0) [0, 22.0]	8.7% (7.2%-10.3%)	9.9% (8.4%-11.7%)	11.2% (9.5%-13.1%)
	Isolated coarctation	9.9 (3.7-15.8) [0, 22.0]	6.7% (5.8%-7.6%)	8.0% (7.0%-9.0%)	10.1% (8.9%-11.3%)
**VSD**	Total (VSD)	9.9 (4.7-15.2) [0, 22.0]	0.4% (0.2%-0.5%)	1.5% (1.2%-1.9%)	2.0% (1.6%-2.4%)

	Multiple VSDs	8.9 (3.7-14.8) [0, 21.9]	1.0% (0.3%-2.3%)	5.5% (3.5%-8.2%)	6.2% (4.1%-9.1%)
	Isolated VSD	9.9 (4.8-15.2) [0, 22.0]	0.3% (0.2%-0.5%)	1.2% (1.0%-1.6%)	1.7% (1.3%-2.0%)

Figure S1: Inclusion and exclusion flow chart

AVSD=atrioventricular septal defect; DORV=double outlet right ventricle; FUH=functionally univentricular heart; HLHS=hypoplastic left heart syndrome; TGA= transposition of the great arteries; VSD=ventricular septal defect.

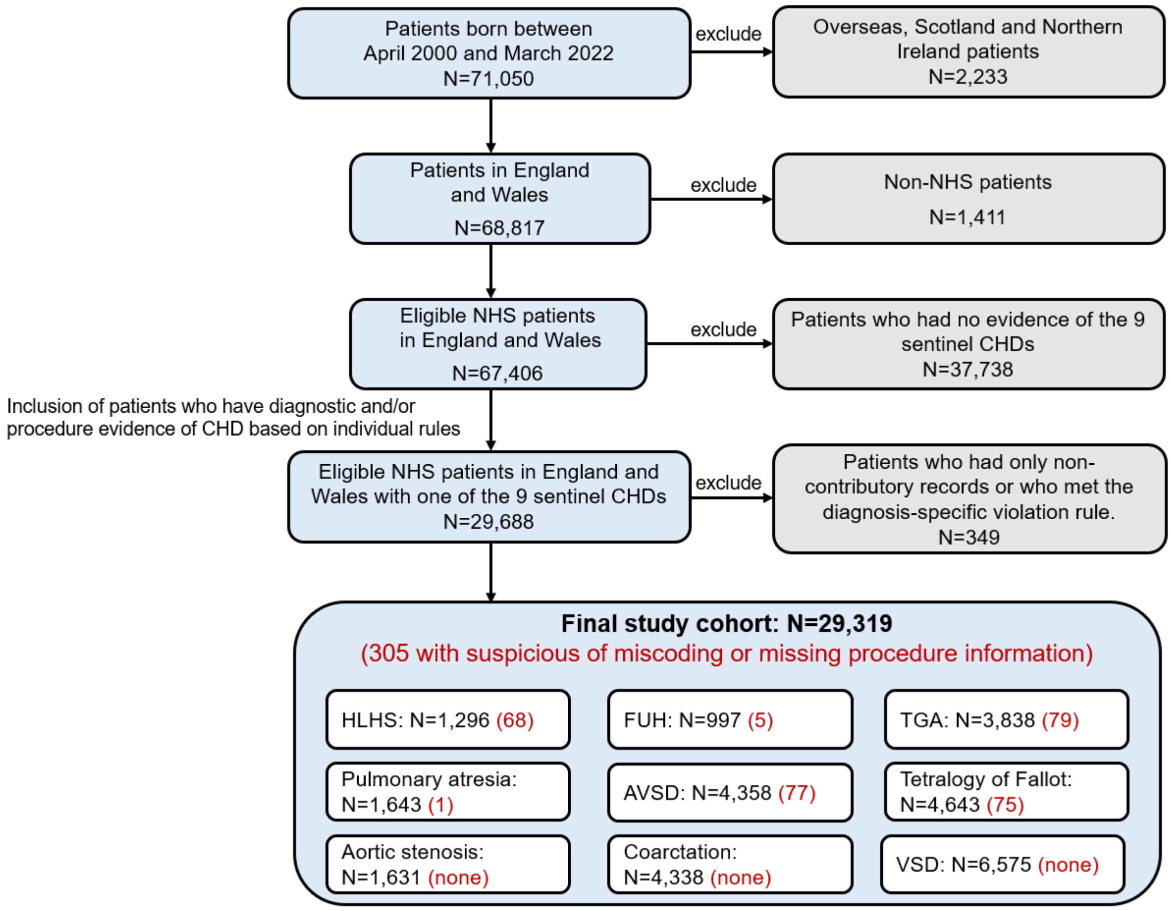

Figure S2: Survival (Kaplan-Meier) curves with 95% confidence interval for subgroups within each CHD diagnosis.

AVSD=atrioventricular septal defect; DORV=double outlet right ventricle; FUH=functionally univentricular heart; HLHS=hypoplastic left heart syndrome; PS=pulmonary stenosis; TGA= transposition of the great arteries; TOF=tetralogy of Fallot; VSD=ventricular septal defect.

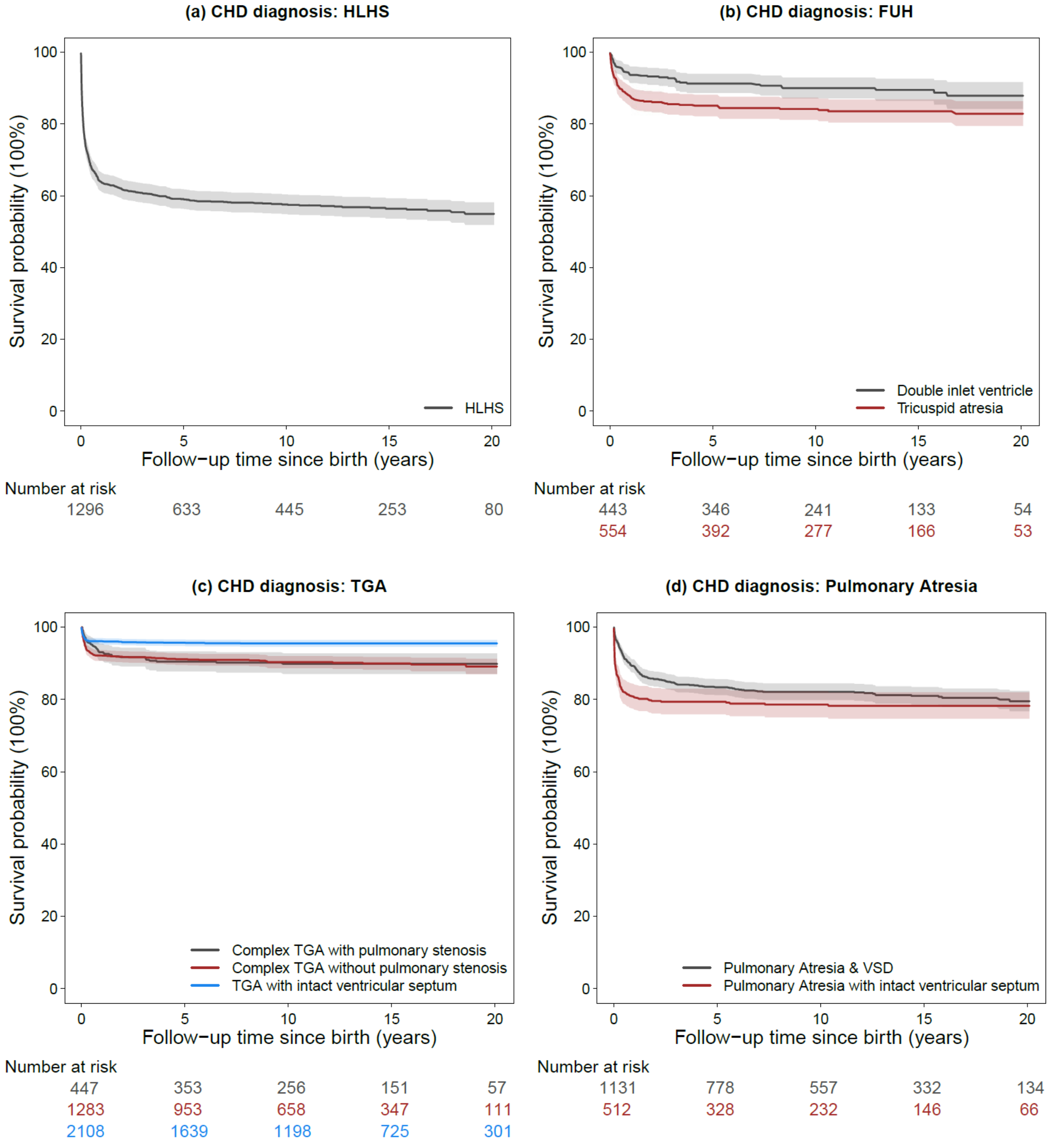

(e) CHD diagnosis: AVSD

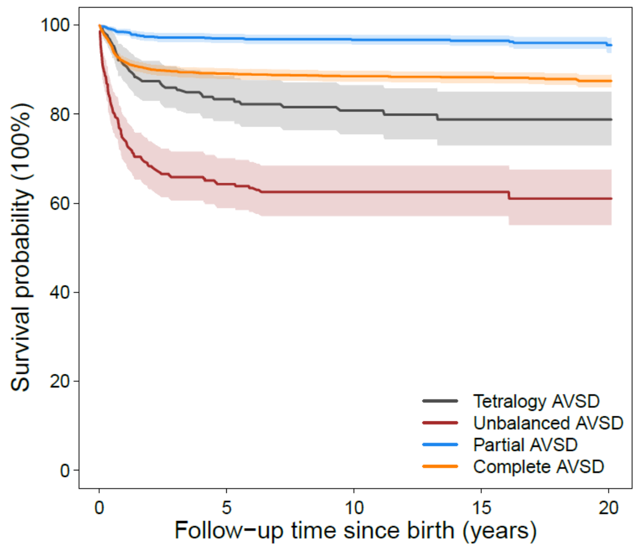

Number at risk

215	152	99	55	14
291	157	103	52	10
1108	980	748	453	183
2744	2016	1427	834	294

(f) CHD diagnosis: TOF

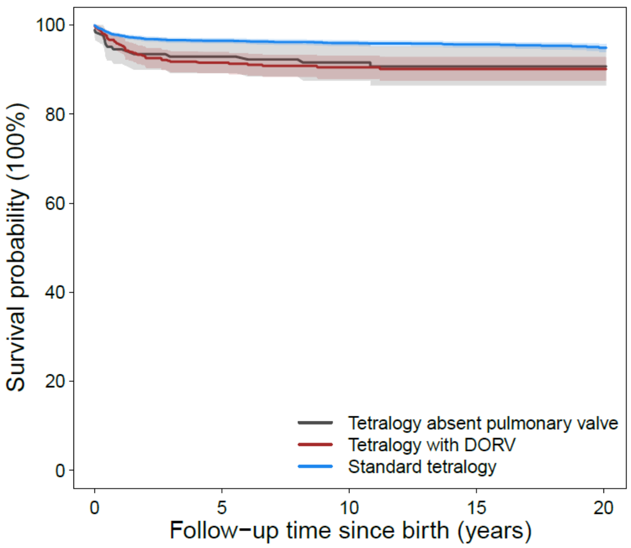

Number at risk

185	157	113	71	30
546	412	256	120	21
3912	3188	2279	1317	535

(g) CHD diagnosis: Aortic Stenosis

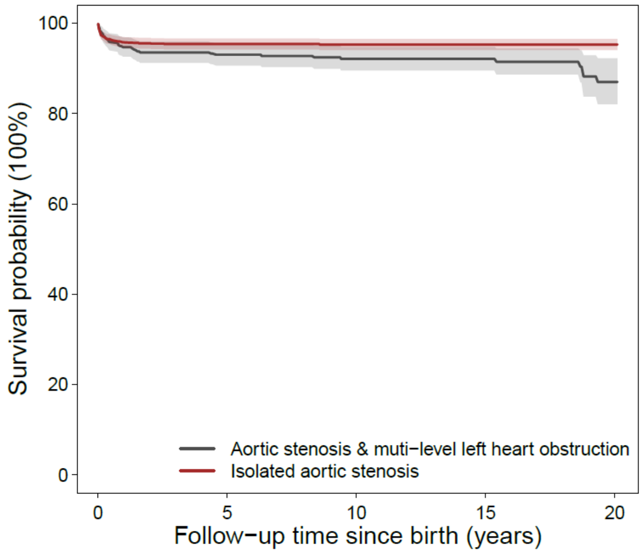

Number at risk

434	352	261	156	56
1197	999	780	496	201

(h) CHD diagnosis: Coarctation

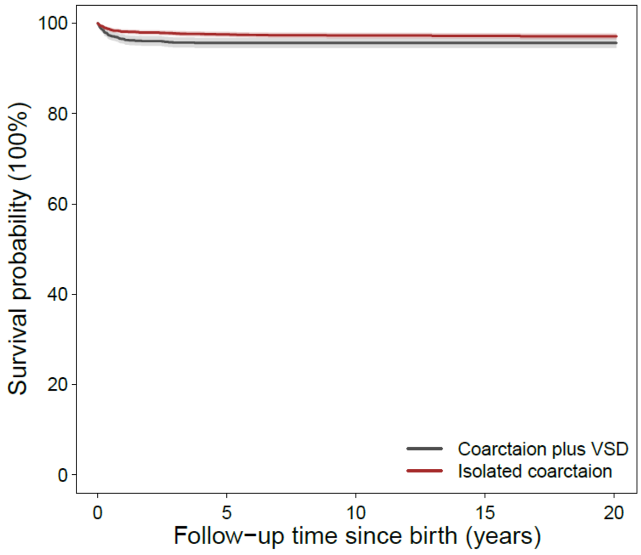

Number at risk

1333	1006	659	362	103
3005	2447	1853	1150	524

(i) CHD diagnosis: VSD

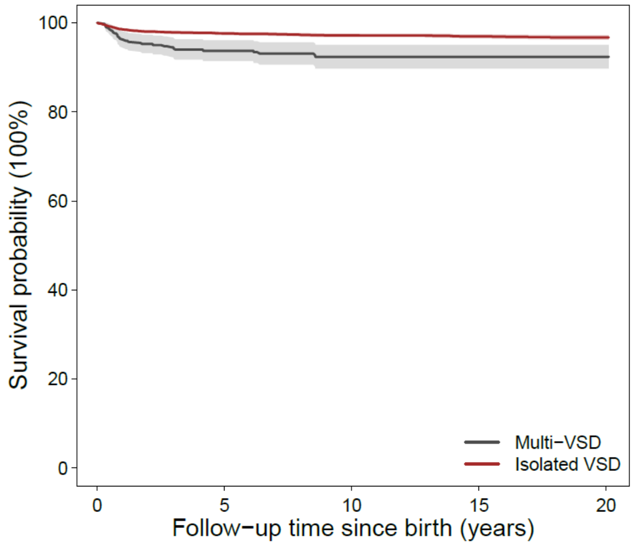

Number at risk

Multi−VSD	426	332	229	133	50
Isolated VSD	6149	5003	3496	2000	747

Figure S3. Cumulative incidence of the first occurrence of reinterventions (any type) with 95% confidence interval for subgroups within each CHD diagnosis.

Death and heart transplant without reintervention were considered as competing events. Patients with suspected missing or miss coded data, 305 (1%), have been removed from the reintervention monitoring.

AVSD=atrioventricular septal defect; DORV=double outlet right ventricle; FUH=functionally univentricular heart; HLHS=hypoplastic left heart syndrome; PS=pulmonary stenosis; TGA= transposition of the great arteries; TOF=tetralogy of Fallot; VSD=ventricular septal defect.

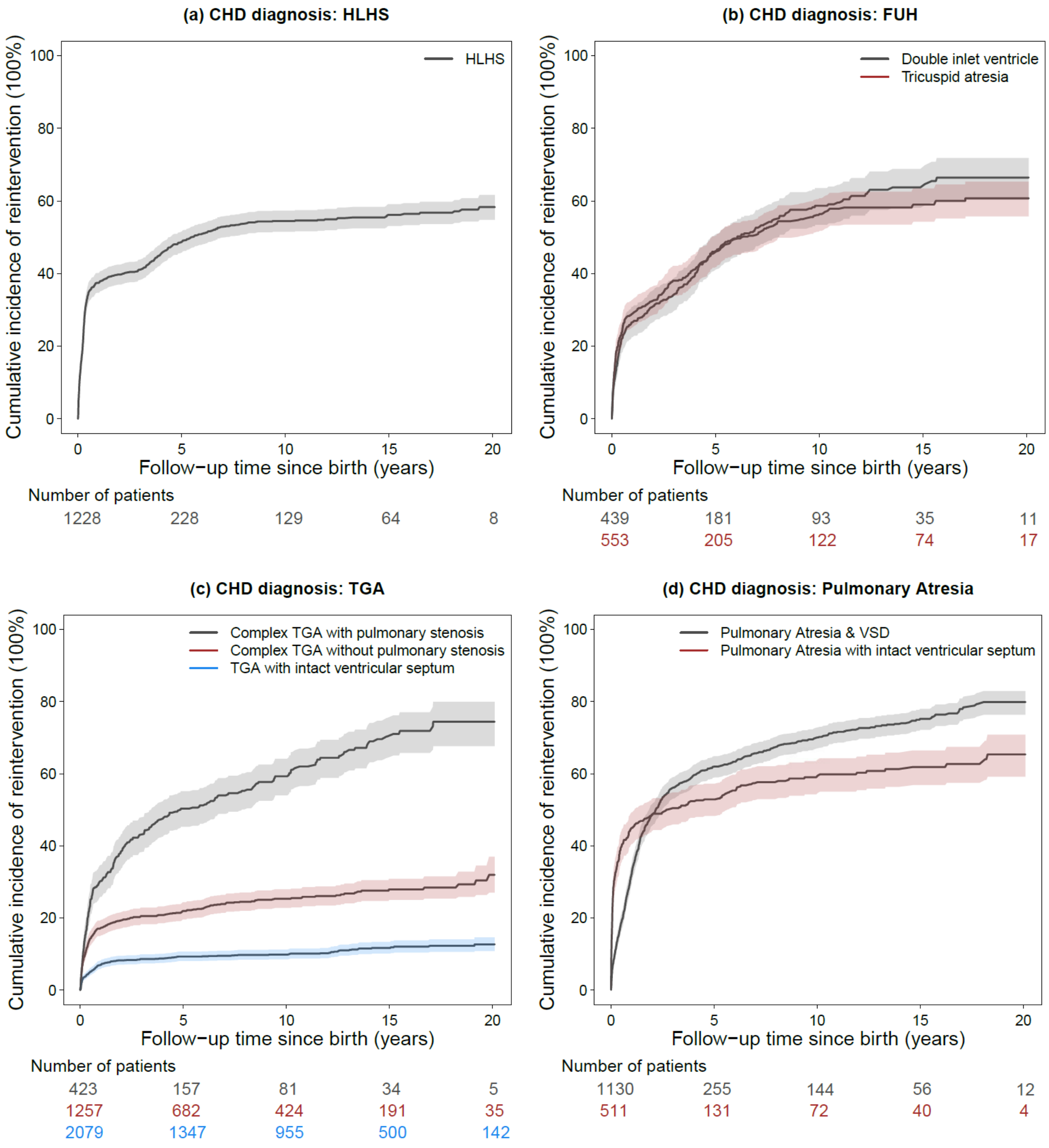

(e) CHD diagnosis: AVSD

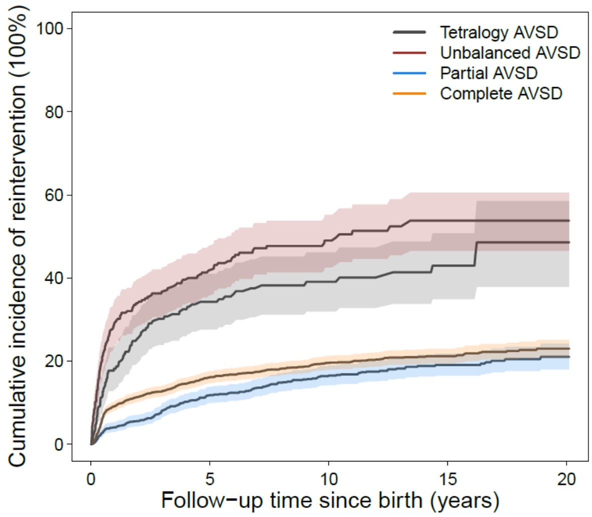

Number of patients

204	92	53	27	6
272	79	40	14	1
1108	841	576	308	80
2697	1576	1024	540	140

(f) CHD diagnosis: TOF

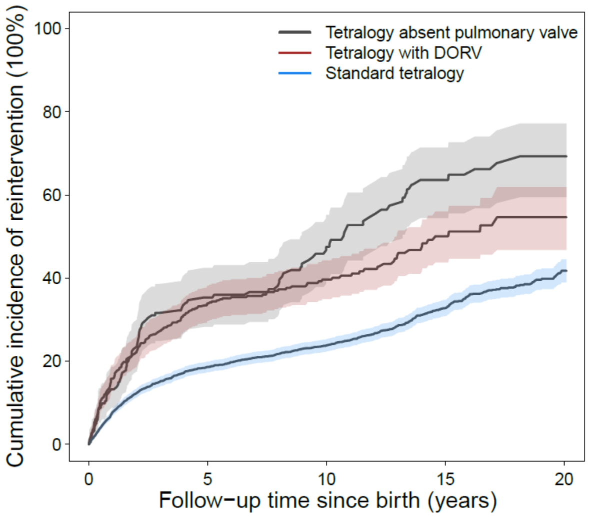

Number of patients

183	97	59	27	11
527	251	127	43	1
3857	2383	1534	715	172

(g) CHD diagnosis: Aortic Stenosis

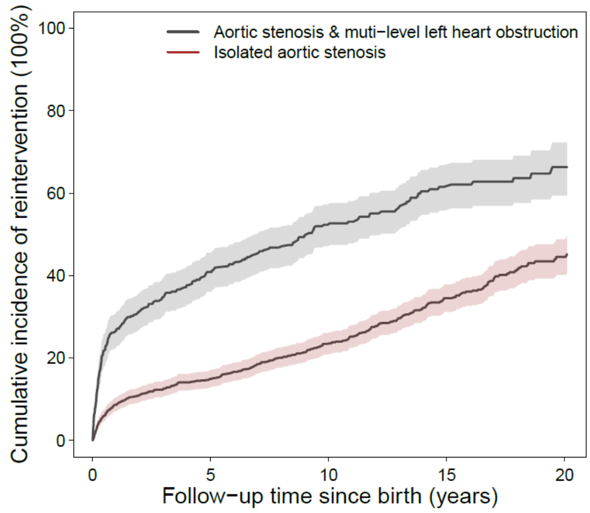

Number of patients

434	211	129	64	15
1197	831	564	292	86

(h) CHD diagnosis: Coarctation

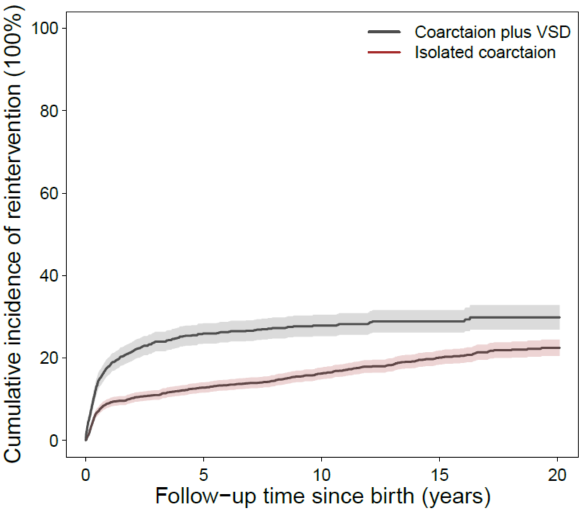

Number of patients

1333	664	412	187	42
3005	2015	1397	771	256

(i) CHD diagnosis: VSD

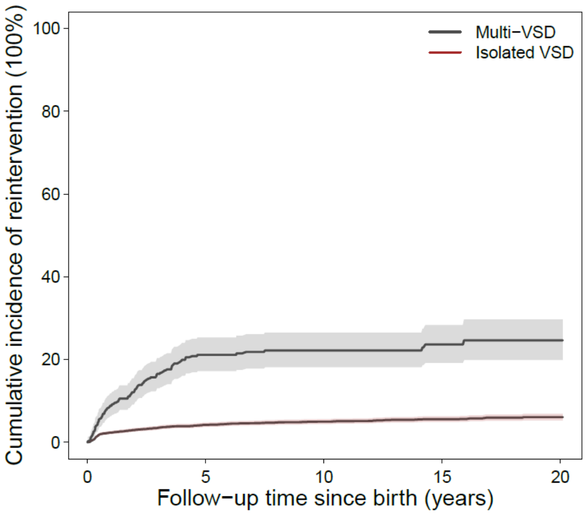

Number of patients

426	231	170	86	20
6147	4423	2936	1536	398
